# Prey fish returned to Forster’s tern colonies suggest spatial and temporal differences in fish composition and availability

**DOI:** 10.1371/journal.pone.0193430

**Published:** 2018-03-15

**Authors:** Sarah H. Peterson, Joshua T. Ackerman, Collin A. Eagles-Smith, Mark P. Herzog, C. Alex Hartman

**Affiliations:** 1 Dixon Field Station, Western Ecological Research Center, U.S. Geological Survey, Dixon, California, United States of America; 2 Forest and Rangeland Ecosystem Science Center, U.S. Geological Survey, Corvallis, Oregon, United States of America; Consejo Superior de Investigaciones Cientificas, SPAIN

## Abstract

Predators sample the available prey community when foraging; thus, changes in the environment may be reflected by changes in predator diet and foraging preferences. We examined Forster’s tern (*Sterna forsteri*) prey species over an 11-year period by sampling approximately 10,000 prey fish returned to 17 breeding colonies in south San Francisco Bay, California. We compared the species composition among repeatedly-sampled colonies (≥ 4 years), using both relative species abundance and the composition of total dry mass by species. Overall, the relative abundances of prey species at seven repeatedly-sampled tern colonies were more different than would be expected by chance, with the most notable differences in relative abundance observed between geographically distant colonies. In general, Mississippi silverside (*Menidia audens*) and topsmelt silverside (*Atherinops affinis*) comprised 42% of individuals and 40% of dry fish mass over the study period. Three-spined stickleback (*Gasterosteus aculeatus*) comprised the next largest proportion of prey species by individuals (19%) but not by dry mass (6%). Five additional species each contributed ≥ 4% of total individuals collected over the study period: yellowfin goby (*Acanthogobius flavimanus*; 10%), longjaw mudsucker (*Gillichthys mirabilis*; 8%), Pacific herring (*Clupea pallasii*; 6%), northern anchovy (*Engraulis mordax*; 4%), and staghorn sculpin (*Leptocottus armatus*; 4%). At some colonies, the relative abundance and biomass of specific prey species changed over time. In general, the abundance and dry mass of silversides increased, whereas the abundance and dry mass of three-spined stickleback and longjaw mudsucker decreased. As central place foragers, Forster’s terns are limited in the distance they forage; thus, changes in the prey species returned to Forster’s tern colonies suggest that the relative availability of some fish species in the environment has changed, possibly in response to alteration of the available habitat.

## Introduction

Quantifying diet and foraging preferences is important for linking birds with their environment and revealing important prey species and their corresponding habitats. Changes in the environment can be reflected by changes in diet, especially among generalists, because animals often sample the available prey community when foraging [1–3]. Human activity and environmental perturbations can alter prey abundance [3–5] and consequently influence prey availability to birds and their diet, which may affect multiple aspects of avian reproduction, offspring survival, and even toxicological risk [6,7]. For example, a reduction in sandeel (*Ammodytes marinus*) and a switch to a species with lower energy content resulted in decreased breeding success of common guillemots (*Uria aalge*) [8].

Bird diet can be challenging to determine, including for adult seabirds provisioning dependent chicks or their mates. Many seabird species nest in locations where direct observation for extended periods is not feasible, expensive, or may cause excessive colony disturbance. Other techniques, such as gastrointestinal tract sampling either involve killing the bird or use of gastric lavage, both of which are invasive. Light stable isotope analysis, using tissues sampled from individual birds, also can be used to estimate diet and foraging behavior [9], but requires the capture and handling of birds. Furthermore, stable isotope analysis typically cannot estimate diet to the level of individual species because multiple prey species may overlap in their isotope values. Sampling excrement or pellets are non-invasive approaches to estimate diet, but are generally biased towards larger prey species with structurally robust hard parts and tend to under represent soft-bodied prey [10]. Furthermore, the prey that adults are consuming may not represent what adults provide to chicks or mates.

Collecting fish that are returned to seabird colonies, and not consumed, during periodic colony visits is minimally invasive, inexpensive, and can reveal the prey selection of adults provisioning chicks [11–13]. The relationship between the type of fish returned to and dropped on the colony and the fish actually consumed by chicks is difficult to ascertain, although three studies on different tern species conducted both direct observations of chick feeding events and collected the fish returned to and dropped on colonies [11–13]. Generally, these studies found similar prey composition between the two approaches, but the two approaches may differ in regards to prey size. Specifically, the proportions of larger bodied prey species were greater in fish that were returned to and dropped on colonies relative to the fish sizes that were consumed by chicks [11–13]. Despite this difference, fish returned to and dropped on colonies can be collected in the same manner among colonies and over time, with minimal disturbance and expense. Thus, this technique is valuable for comparison of prey composition among colonies and changes in prey composition within colonies over time.

Forster’s terns (*Sterna forsteri*) are a primarily piscivorous species of seabird that breeds on small islands and within marshes in North America [14]. Of the Pacific Coast population of Forster’s terns, 30% nest within San Francisco Bay within managed ponds adjacent to the bay [14,15], which also provide critical foraging habitat for the terns [16–18]. Breeding Forster’s terns tend to forage within 6.2 km of their breeding colony [19]; consequently, the prey available to breeding Forster’s terns comes from a restricted geographic area around the colony. Changes to the available prey assemblage over time, due to large-scale regional habitat restoration (www.southbayrestoration.org) or ecological shifts in the managed pond habitats, could influence tern foraging. A major component of regional restoration involves conversion of former salt evaporation ponds to tidal marsh habitat, which may alter the fish species composition in the habitats adjacent to tern colonies [20,21]. Furthermore, mercury concentrations in the potential prey of Forster’s terns in San Francisco Bay varies substantially among species [7]. Consequently, shifts in prey availability could influence the bioaccumulation of mercury by Forster’s terns.

We examined collections of prey fish that were returned to, and dropped at, 17 Forster’s tern colonies in San Francisco Bay over an 11-year period. We determined 1) if the species composition of prey items returned to tern colonies varied among colonies and 2) whether the relative abundance, relative mass, and size of the most common prey items changed over time.

## Methods

### Sample collection

**Fig 1:**
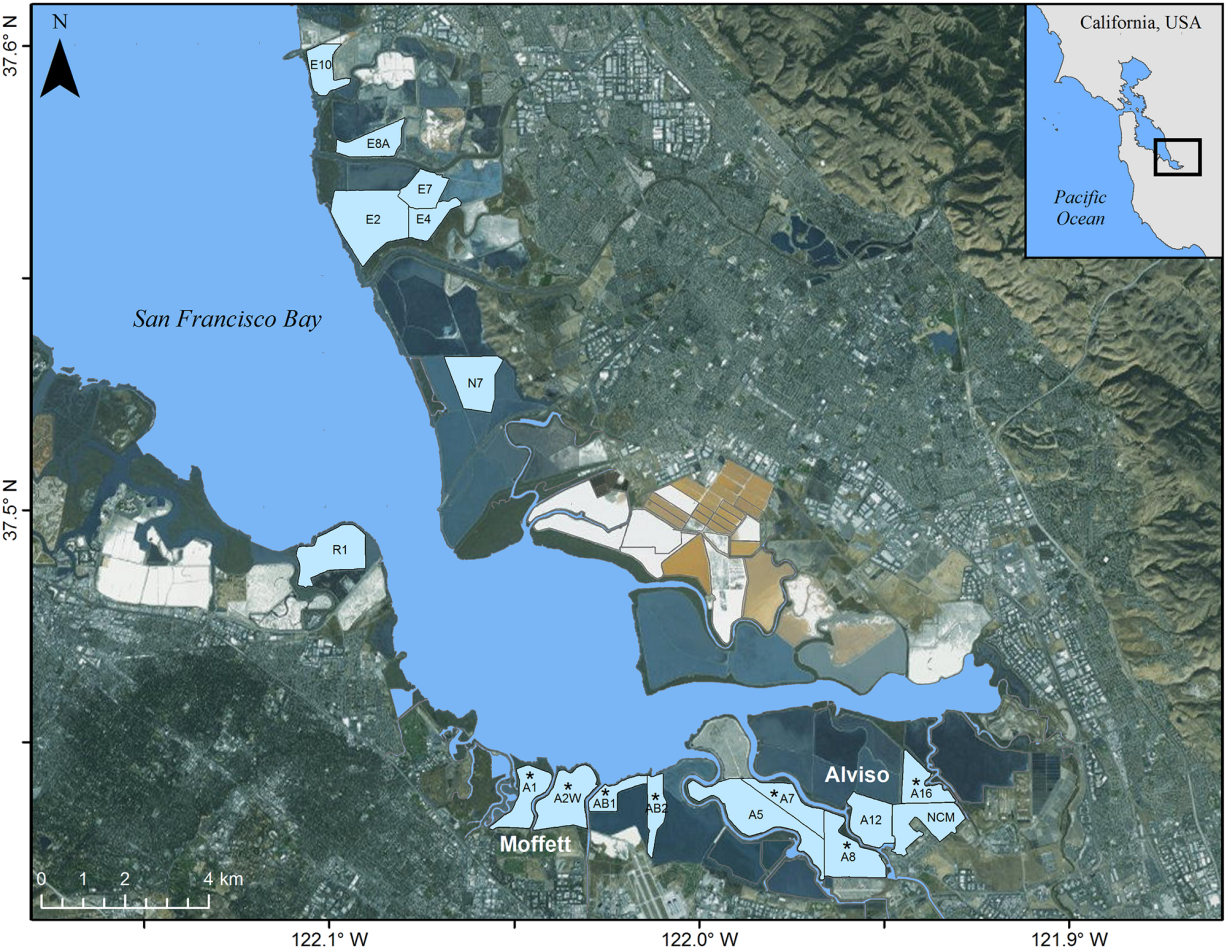
Fish returned to 17 Forster’s tern (*Sterna forsteri*) breeding colonies in south San Francisco Bay. Fish were collected during weekly nest-monitoring visits during 2005–2015 (April through September while the colony was active). Terns did not nest at every colony in every year. Colonies included in statistical analyses are indicated by an * (sampled in ≥ 4 years with ≥ 25 fish collected/year). Colonies in the southern-most portion of the bay are separated into Moffett (A1, A2W, AB1, and AB2) and Alviso regions (A5-A16 and NCM). Imagery Service Layer Credits: Source: Esri, Digital Globe, GeoEye, Earthstar Geographics, CNES/Airbus DS, USDA, USGS, AEX, Getmapping, Aerogrid, IGN, IGP, swisstopo, and the GIS User Community.

From 2005 to 2015, we monitored up to 17 Forster’s tern colonies (hereafter colonies) in the southern San Francisco Bay Estuary, mainly located on islands within managed ponds (Fig 1), and entered colonies weekly to monitor nests and chicks [22]. We collected samples each time a colony was visited, as early as April 17 and as late as Sept 19, depending on when the colonies were active during each year. We refer to the colonies by the name of the pond in which they were located, and in some cases a colony was comprised of several adjacent islands within the same pond. Most tern colonies within the southern San Francisco Bay Estuary were monitored each year. The colony locations of terns varied annually; therefore, some islands were used during most years whereas other islands may have been used during only one year.

During weekly colony visits, we searched for any fish or invertebrates that were on the ground in the colony. Forster’s terns carry prey back to the colony to feed to their chicks or deliver to their mates [23]. Periodically the prey are dropped on the ground prior to feeding or the chick may reject the prey. Because Forster’s terns are the only piscivorous species nesting within these breeding colonies, apart from an occasional (< five nests in south San Francisco Bay per year) black skimmer (*Rynchops niger*) nest, we are confident that the majority of fish returned to the Forster’s tern colony were brought back by Forster’s terns. We collected all fish and other potential prey items found dropped in the colony during each weekly nest visit and stored samples in reclosable plastic bags. One period of samples from the A16 colony was excluded because it coincided with a major fish kill due to temporarily low dissolved oxygen concentrations in the pond, and because other piscivorous birds were known to be roosting on those islands at that time [24]. Forster’s terns are primarily piscivorous [14,23,25], although invertebrates also have been documented to be consumed [23]. We recognize that our sampling was biased against invertebrates due to their smaller size and because invertebrates may decompose faster than fish; although we collected them when they were observed. However, invertebrates comprised < 0.1% of all collected samples and the remainder were fish; therefore, we removed invertebrates from all analyses. We stored samples frozen at -20°C until they could be processed in the laboratory and dried.

In the laboratory, we identified prey samples to the lowest taxonomic group. Most fish were identified to species although some could only be identified to family. In particular, Mississippi silverside (*Menidia audens*) and topsmelt silverside (*Atherinops affinis*) from the Atherinopsidae family sometimes could not be separated because fish were typically desiccated, which prohibited the use of some identification marks. For example only 5.4% of all Atherinopsidae were identified to species in 2011, whereas > 99% of Atherinopsidae were identified to species in 2005 and 2007. As a result, we combined all Atherinopsidae into one group, silversides, for statistical analyses. We present scientific names of all identified species or the lowest identified taxonomic group in Table 1.

**Table 1:** Relative abundance of fish returned to Forster’s tern (*Sterna forsteri*) colonies in south San Francisco Bay during 2005–2015, calculated for each colony and year and all colonies together.

			Relative abundance per species										
Colony	Year	n	Family Atherinopsidae (*Menidia audens* and *Atherinops affis*)	Family Embiotocidae	Longjaw mudsucker (*Gillichthys mirabilis*)	Northern anchovy (*Engraulis mordax*)	Pacific herring (*Clupea pallasii*)	Rainwater killifish (*Lucania parva*)	Staghorn sculpin (*Leptocottus armatus*)	Three-spined stickleback (*Gasterosteus aculeatus*)	Yellowfin goby (*Acanthogobius flavimanus*)	Other gobies^a^	Other^b^
A1*	2005	176	35.8	3.4	1.1	20.5	2.3	1.1	1.7	4.0	11.4	11.9	6.8
A1*	2007	393	56.0	0.5	3.1	3.1	1.3	1.5	1.8	19.1	12.2	0.0	1.5
A1*	2008	595	52.9	0.0	0.5	4.2	27.6	0.5	5.0	5.2	2.5	0.5	1.0
A1*	2009	134	76.9	0.7	3.0	3.0	0.0	7.5	0.7	3.7	2.2	0.0	2.2
A1*	2010	76	55.3	0.0	0.0	5.3	0.0	0.0	3.9	13.2	22.4	0.0	0.0
A1*	2011	96	58.3	1.0	0.0	5.2	6.3	2.1	5.2	1.0	17.7	0.0	3.1
A1	2012	8	50.0	0.0	0.0	0.0	25.0	0.0	12.5	0.0	12.5	0.0	0.0
A1	2013	23	60.9	4.3	0.0	0.0	17.4	0.0	8.7	0.0	0.0	0.0	8.7
A1	2014	1	100.0	0.0	0.0	0.0	0.0	0.0	0.0	0.0	0.0	0.0	0.0
A5	2007	275	73.5	0.0	0.7	1.5	0.4	0.7	0.0	22.9	0.0	0.0	0.4
A7	2005	19	5.3	5.3	15.8	5.3	0.0	0.0	10.5	0.0	0.0	5.3	52.6
A7*	2006	615	14.3	0.2	21.0	0.7	4.7	1.0	0.7	33.5	7.5	2.8	13.8
A7*	2007	184	37.5	0.0	4.9	0.5	2.2	0.5	0.0	45.7	8.7	0.0	0.0
A7*	2008	264	32.6	2.7	8.3	3.0	24.6	0.8	1.5	23.1	2.7	0.4	0.4
A7*	2009	150	44.7	3.3	20.0	4.7	3.3	2.7	6.0	2.0	6.0	5.3	2.0
A7*	2010	315	19.0	0.0	7.6	1.0	1.9	0.3	10.8	26.7	26.3	6.3	0.0
A7*	2011	134	27.6	0.0	14.2	1.5	5.2	0.0	8.2	4.5	35.1	2.2	1.5
A7*	2012	38	42.1	0.0	15.8	2.6	2.6	0.0	2.6	26.3	5.3	0.0	2.6
A7	2013	9	88.9	0.0	0.0	0.0	11.1	0.0	0.0	0.0	0.0	0.0	0.0
A8	2005	14	57.1	7.1	14.3	0.0	7.1	0.0	0.0	14.3	0.0	0.0	0.0
A8*	2006	37	40.5	0.0	16.2	0.0	0.0	0.0	0.0	8.1	8.1	0.0	27.0
A8	2007	1	100.0	0.0	0.0	0.0	0.0	0.0	0.0	0.0	0.0	0.0	0.0
A8	2008	19	26.3	0.0	10.5	0.0	42.1	0.0	0.0	15.8	5.3	0.0	0.0
A8	2009	11	72.7	0.0	0.0	0.0	9.1	0.0	18.2	0.0	0.0	0.0	0.0
A8*	2010	59	42.4	0.0	10.2	1.7	5.1	0.0	8.5	0.0	32.2	0.0	0.0
A8	2011	16	68.8	0.0	0.0	0.0	6.3	0.0	18.8	0.0	6.3	0.0	0.0
A8*	2012	25	52.0	0.0	4.0	0.0	16.0	0.0	0.0	28.0	0.0	0.0	0.0
A8*	2013	86	39.5	0.0	0.0	8.1	34.9	2.3	0.0	4.7	4.7	0.0	5.8
A12	2008	7	14.3	0.0	14.3	0.0	28.6	0.0	28.6	14.3	0.0	0.0	0.0
A16*	2005	160	49.4	3.1	13.8	0.0	0.0	0.0	0.0	0.0	0.0	27.5	6.3
A16	2006	17	64.7	0.0	11.8	0.0	23.5	0.0	0.0	0.0	0.0	0.0	0.0
A16*	2007	279	43.7	0.4	5.4	1.4	2.5	1.4	2.2	26.9	15.8	0.0	0.4
A16*	2008	1388	11.7	0.5	18.4	3.4	3.5	2.2	3.0	43.4	12.4	0.9	0.7
A16*	2009	501	33.9	0.8	18.2	1.8	1.4	2.8	3.0	24.8	9.0	3.4	1.0
A16*	2010	163	16.0	0.0	15.3	1.8	2.5	1.2	5.5	46.0	11.7	0.0	0.0
A16	2011	4	0.0	0.0	50.0	0.0	50.0	0.0	0.0	0.0	0.0	0.0	0.0
A2W	2008	3	100.0	0.0	0.0	0.0	0.0	0.0	0.0	0.0	0.0	0.0	0.0
A2W*	2009	238	64.3	0.8	7.6	7.6	0.8	0.4	5.5	4.2	5.9	2.1	0.8
A2W*	2010	134	33.6	0.7	1.5	1.5	4.5	0.7	2.2	11.2	42.5	0.7	0.7
A2W*	2011	87	50.6	0.0	2.3	2.3	12.6	0.0	6.9	3.4	21.8	0.0	0.0
A2W*	2012	156	55.8	0.6	1.3	8.3	6.4	1.9	3.8	12.8	8.3	0.0	0.6
A2W*	2013	241	56.4	1.7	0.0	7.1	12.9	0.4	7.1	2.1	10.0	0.0	2.5
A2W*	2014	173	85.5	0.6	0.6	2.3	1.7	1.7	0.0	0.6	5.8	0.0	1.2
A2W*	2015	147	91.8	0.0	0.0	0.0	0.0	0.7	0.7	0.0	6.1	0.0	0.7
AB1*	2008	564	35.3	2.7	2.3	7.6	5.5	0.0	11.9	23.9	10.1	0.0	0.7
AB1*	2010	267	33.7	0.7	3.4	1.9	1.9	0.0	13.9	3.7	39.0	1.5	0.4
AB1	2011	1	0.0	0.0	0.0	0.0	100.0	0.0	0.0	0.0	0.0	0.0	0.0
AB1*	2012	174	58.0	0.0	1.7	16.1	6.9	1.1	2.3	6.9	5.2	0.0	1.7
AB1*	2013	67	40.3	1.5	0.0	10.4	34.3	0.0	4.5	6.0	0.0	0.0	3.0
AB1*	2014	39	87.2	2.6	0.0	7.7	2.6	0.0	0.0	0.0	0.0	0.0	0.0
AB1*	2015	236	76.3	0.8	1.3	4.7	0.0	0.4	0.0	0.0	13.6	0.8	2.1
AB2	2008	1	0.0	0.0	0.0	0.0	0.0	0.0	100.0	0.0	0.0	0.0	0.0
AB2*	2009	34	41.2	14.7	2.9	17.6	0.0	0.0	5.9	8.8	0.0	2.9	5.9
AB2	2010	3	33.3	0.0	0.0	0.0	0.0	0.0	0.0	66.7	0.0	0.0	0.0
AB2	2012	9	55.6	0.0	11.1	0.0	0.0	0.0	11.1	11.1	11.1	0.0	0.0
AB2*	2013	64	76.6	0.0	0.0	4.7	3.1	0.0	6.3	4.7	1.6	0.0	3.1
AB2*	2014	193	89.6	0.0	0.5	1.0	0.0	0.0	3.6	0.0	5.2	0.0	0.0
AB2*	2015	35	74.3	0.0	0.0	5.7	0.0	0.0	0.0	0.0	20.0	0.0	0.0
E2	2010	100	84.0	1.0	3.0	4.0	0.0	0.0	6.0	0.0	2.0	0.0	0.0
E2	2012	57	43.9	0.0	0.0	1.8	3.5	47.4	0.0	0.0	3.5	0.0	0.0
E4	2007	5	80.0	0.0	0.0	0.0	0.0	0.0	20.0	0.0	0.0	0.0	0.0
E7	2007	85	60.0	2.4	4.7	7.1	3.5	2.4	1.2	7.1	9.4	0.0	2.4
E7	2009	2	50.0	0.0	0.0	50.0	0.0	0.0	0.0	0.0	0.0	0.0	0.0
E7	2012	2	100.0	0.0	0.0	0.0	0.0	0.0	0.0	0.0	0.0	0.0	0.0
E8A	2005	20	75.0	5.0	0.0	5.0	10.0	0.0	0.0	0.0	5.0	0.0	0.0
E10	2014	76	36.8	25.0	0.0	9.2	0.0	3.9	10.5	3.9	10.5	0.0	0.0
NCM	2006	1	0.0	0.0	100.0	0.0	0.0	0.0	0.0	0.0	0.0	0.0	0.0
NCM	2010	1	0.0	100.0	0.0	0.0	0.0	0.0	0.0	0.0	0.0	0.0	0.0
NCM	2013	10	20.0	0.0	0.0	0.0	10.0	0.0	10.0	0.0	40.0	0.0	20.0
NCM	2014	5	60.0	0.0	0.0	20.0	0.0	0.0	0.0	0.0	0.0	0.0	20.0
NCM	2015	48	83.3	0.0	2.1	0.0	0.0	0.0	0.0	8.3	6.3	0.0	0.0
N7	2006	270	24.8	0.4	22.6	1.5	1.5	1.5	1.1	28.1	0.7	3.3	14.4
R1	2009	137	52.6	0.0	5.8	29.2	0.0	3.6	1.5	0.0	5.1	1.5	0.7
R1	2010	1	100.0	0.0	0.0	0.0	0.0	0.0	0.0	0.0	0.0	0.0	0.0
All	2005	389	42.7	3.6	7.5	9.8	1.8	0.5	1.3	2.3	5.4	17.0	8.2
All	2006	940	19.3	0.2	21.2	0.9	3.9	1.1	0.7	30.3	5.4	2.8	14.3
All	2007	1222	54.7	0.4	3.4	2.2	1.6	1.2	1.2	24.8	9.5	0.0	0.8
All	2008	2841	27.1	1.0	10.5	4.3	11.2	1.2	5.1	29.3	8.9	0.6	0.7
All	2009	1207	48.7	1.4	12.6	7.0	1.2	2.8	3.6	12.0	6.5	2.7	1.3
All	2010	1119	33.4	0.4	6.2	2.0	2.1	0.4	8.7	17.5	26.9	2.2	0.2
All	2011	338	43.8	0.3	6.8	2.7	8.3	0.6	7.4	3.0	24.9	0.9	1.5
All	2012	469	53.9	0.2	2.8	9.2	6.6	6.8	2.8	10.7	6.0	0.0	1.1
All	2013	500	54.0	1.2	0.0	6.8	18.4	0.6	5.4	3.2	6.6	0.0	3.8
All	2014	487	79.5	4.3	0.4	3.5	0.8	1.2	3.1	0.8	5.7	0.0	0.6
All	2015	466	81.8	0.4	0.9	2.8	0.0	0.4	0.2	0.9	10.9	0.4	1.3
All	All	9978	42.0	1.0	8.3	4.2	5.8	1.5	4.0	18.6	10.5	1.7	2.5

^a^ Other gobies include arrow goby (*Clevelandia ios*) and any unidentified *Acanthogobius* species.

^b^ Other fish species included black crappie (*Pomoxis nigromaculatus*), bluegill (*Lepomis macrochirus*), California roach (*Hesperoleucus symmetricus*), common carp (*Cyprinus carpio*), largemouth bass (*Micropterus salmoides*), bay pipefish (*Syngnathus leptorhynchus*), prickly sculpin (*Cotter asper*), salmonids (Genus *Oncorhynchus*), starry flounder (*Platichthys stellatus*), and western mosquitofish (*Gambusia affinis*).

^c^ Indicates repeatedly-sampled colonies with ≥ 25 samples collected within a year that were included in statistical analyses.

We gently cleaned prey samples using deionized water. We measured standard length of each fish to the nearest mm. Fish were placed in individual containers and dried at 50°C for approximately 24–48 hr. After drying, we weighed each fish to obtain a dry mass and measured standard length again to obtain a dry standard length. When individual fish were missing large portions of their body (approximately ≥ 5%), we substituted a species- and year-specific mean dry mass for those individual fish. Similarly, if we could not obtain an accurate standard length of the dry fish, we substituted the species- and year-specific mean standard length of dried fish. Forster’s terns have been observed to consume fish 10–100 mm in length, with the majority (81%) of freshly-caught fish 50–70 mm in size [25]. For comparison with previous studies on Forster’s terns and other tern species, we assigned fish to one of five dry standard length categories based on Atwood and Kelly (1984): < 25 mm, 25–50 mm, 50–75 mm, 75–100 mm, and > 100 mm.

### Statistical analyses

We quantified species composition in two ways, one based on species counts (hereafter relative species abundance) and one based on total dry mass of fish species (hereafter dry mass composition), at each tern colony for each year. Then we used a subset of colonies that we repeatedly sampled over time to test whether the relative species abundance and dry mass composition of prey items differed among colonies. Furthermore, we used the same subset of repeatedly sampled colonies to test whether species’ relative abundances changed over the duration of our 11-year study. For these tests, we only used those tern colonies that we sampled repeatedly over the course of the study (during 4–7 different years) and had ≥ 25 fish collected per year.

To compare relative species abundance and dry mass composition among colonies, we used a multi-response permutation procedure (mrpp; [26]) in the *vegan* package [27] with the statistical program R version 3.3.2 [28]. This statistical approach compares dissimilarities within and among groups to tell whether there are significant differences between groups. We used the Bray-Curtis distance measure to calculate the dissimilarity matrix used by mrpp, in order to determine and test within-group similarity and distinctness [29]. To compare species composition among colonies, we used relative species abundance to calculate the Bray-Curtis distance measure, which controlled for differences in the total number of fish collected at each tern colony per year. Similarly, for dry mass composition, we used the proportion of dry mass per species, which controlled for differences in the total fish mass collected at each tern colony per year. If relative species abundance or the dry mass composition differed among tern colonies, we conducted individual mrpp analyses between all pairs of colonies. Mrpp analysis calculates a δ value between and within groups, which is the weighted mean within-group distance [26]. Mrpp analysis also provides an *A* statistic, that describes the effect size of the grouping, and a *p* value, that quantifies the likelihood that the observed difference is due to chance [26,30]. The *A*-statistic can be interpreted similarly to the coefficient of determination in a linear model [27]. If all samples within groups are identical then *A* = 1. Conversely, *A* = 0 if within-group heterogeneity equals what is expected by chance [26].

To examine trends in the relative species abundance and dry mass composition of prey items over an 11-year sampling period during the Forster’s tern breeding season, we started globally with a multivariate general linear model (MANOVA) for the proportions of the most abundant prey species, as a function of year, colony, and a year × colony interaction effect. We included the seven species that each comprised > 4% of all samples as dependent variables, with one value per species for every year and colony combination. The year × colony interaction effect provided the ability to test for overall differences in slope coefficients among colonies. After running the global MANOVA, we ran individual univariate linear models for each species with the same fixed effects to determine the source of the significance at the global level. If the interaction term for a specific species was significant (*p* < 0.05), we compared colonies using slope coefficients, standard errors, and 95% confidence intervals produced using the *lsmeans* package in R [31]. If the interaction term for a specific species was not significant (*p* > 0.05), we dropped the interaction term from the model. For models without a significant interaction effect, we examined pairwise differences in model-generated least squares mean relative abundances if there was a significant colony effect. We used a logit transformation on all proportional data prior to analysis, with the lowest non-zero value (≤ 0.005) as a substitute for all zeros in our dataset [32]. We present the differences in relative abundance among colonies from back-transformed least squares means for the average year.

We used linear mixed effects models to examine whether dry standard length or dry mass of the seven most commonly observed fish species changed over the course of our study at the same repeatedly sampled colonies that we used to examine relative abundance and dry mass composition over time. We excluded fish from these analyses that were missing standard length measurements or large portions of their body (≥ 5%). We examined each fish species separately for standard length and log-transformed mass. For each species, we first compared three models with year as a fixed effect and different random effects to determine whether our data were best explained by 1) a random intercept and slope model that allowed both the intercept and slope to vary by colony, 2) a random intercept model that allowed the intercept to vary by colony, or 3) a model with no random terms. We fit each model using restricted maximum likelihood (REML) and used the AIC values to select the best model. The best models to examine temporal trends of fish size for all seven species included a random intercept for colony. The only exception was for standard length of staghorn sculpin, where the best model did not include random effects. Therefore, we present only the results for the best models. We used the *afex* R package to determine significance with *F* tests, using the Kenward-Roger approximation for degrees of freedom [33].

## Results

### Relative species abundance, dry mass composition, and dry fish standard length

**Fig 2:**
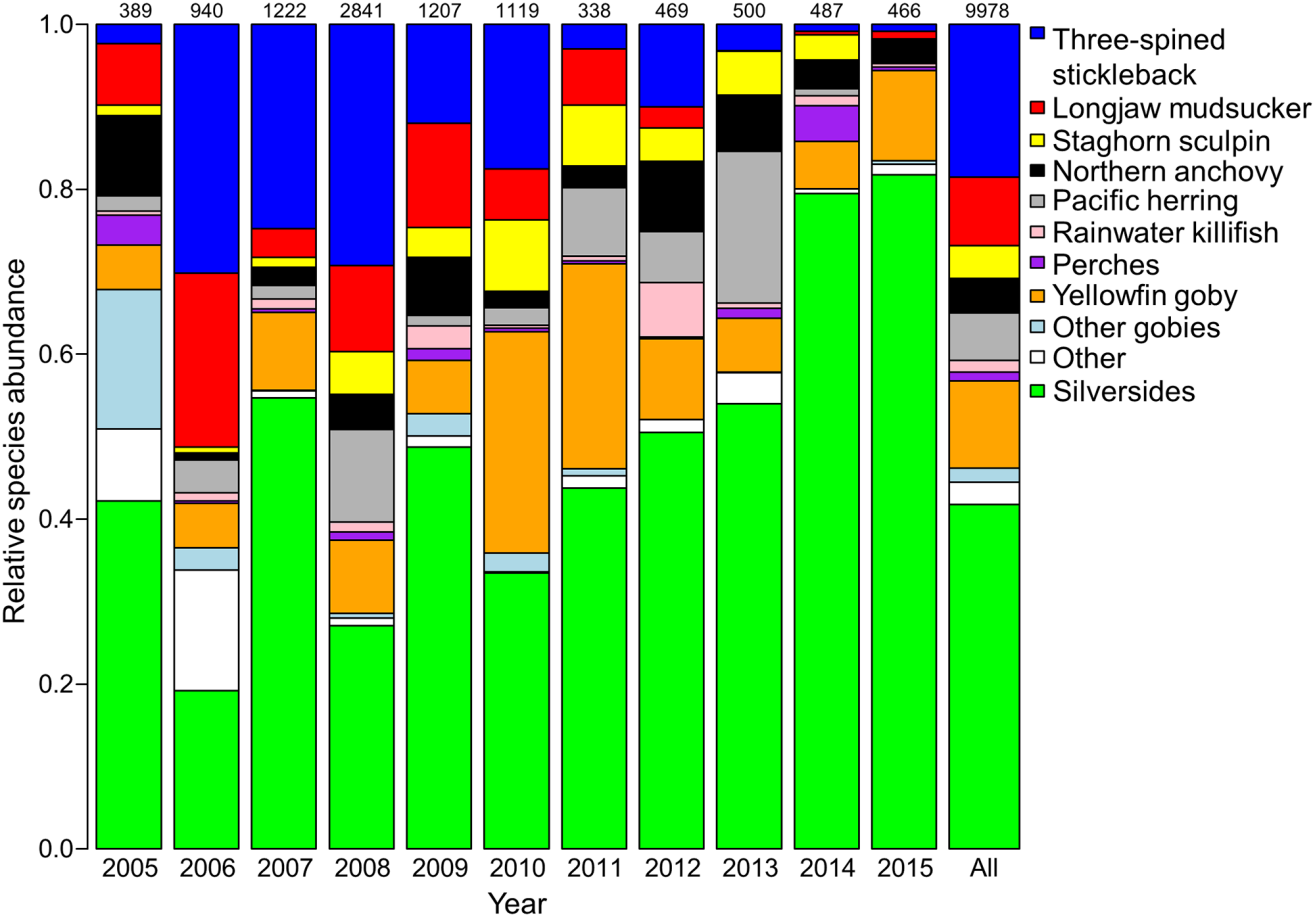
Relative abundance of fish returned to Forster’s tern (*Sterna forsteri*) colonies. Prey fish were collected at tern breeding colonies in south San Francisco Bay, California during 2005–2015. The total number of individuals collected within a year are shown above the bars. The other category includes additional species of fish. The bars are presented in the same order as the legend.

We collected 9,978 fish samples from 17 Forster’s tern colonies in San Francisco Bay between 2005 and 2015 (Table 1). The annual abundance of dropped fish at all tern colonies ranged from a minimum of 338 in 2011 to a maximum of 2,841 in 2008 (Fig 2). Overall, silversides were the most abundant fish returned to tern colonies, representing 42.0% of individuals over the 11-year period. Three-spined stickleback (*Gasterosteus aculeatus*) were the second most abundant species (18.6%) followed by yellowfin goby (*Acanthogobius flavimanus*; 10.5%), longjaw mudsucker (*Gillichthys mirabilis*; 8.3%), Pacific herring (*Clupea pallasii*; 5.8%), northern anchovy (*Engraulis mordax*; 4.2%), and staghorn sculpin (*Leptocottus armatus*; 4.0%). Other species of goby (*Acanthogobius* spp), rainwater killifish (*Lucania parva*), and perch species (family Embiotocidae) each comprised < 2% of total fish collected. Additional fish species and fish that were unable to be identified, comprised 2.5% of total individuals collected. Over the course of the study, other fish species that were identified at tern colonies included black crappie (*Pomoxis nigromaculatus*; *n* = 6), bluegill (*Lepomis macrochirus*; *n* = 2), California roach (*Hesperoleucus symmetricus*; *n* = 1), common carp (*Cyprinus carpio*; *n* = 2), largemouth bass (*Micropterus salmoides*; *n* = 27), bay pipefish (*Syngnathus leptorhynchus*; *n* = 8), prickly sculpin (*Cotter asper*; *n* = 18), salmonids (Genus *Oncorhynchus*; *n* = 2), starry flounder (*Platichthys stellatus*; *n* = 28), and western mosquitofish (*Gambusia affinis*; *n* = 2; Table 1).

**Fig 3:**
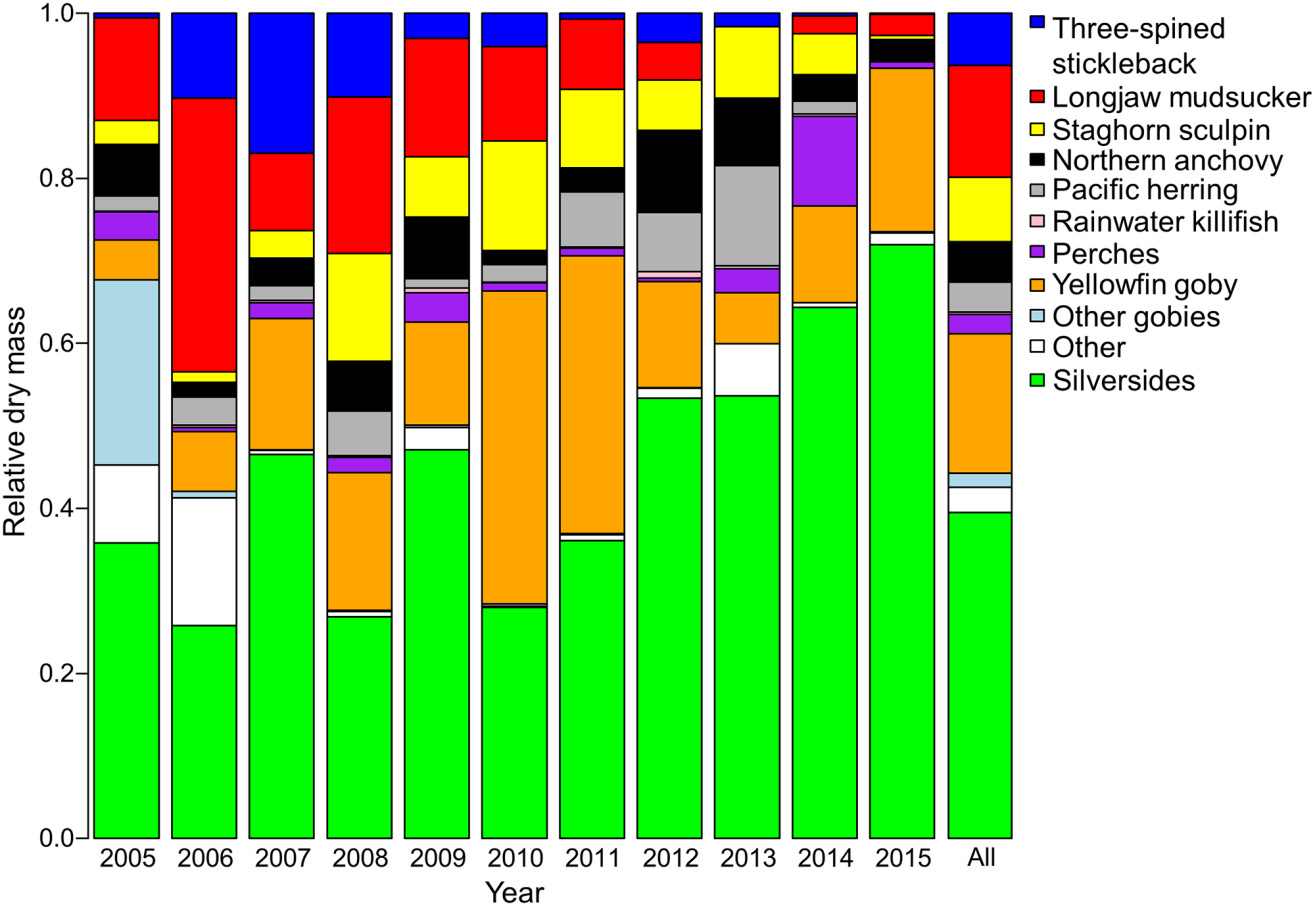
Relative dry mass by fish species returned to Forster’s tern (*Sterna forsteri*) colonies. Prey fish were collected at tern breeding colonies in south San Francisco Bay, California during 2005–2015. The total number of individuals collected within a year is shown above the bars. The other category includes additional species of fish. The bars are presented in the same order as the legend.

Similar to relative species abundance, silversides comprised the largest proportion of dry fish mass (39.7%); however, the proportional contribution of the remaining groups differed between dry mass composition and their relative abundances (Figs 2 and 3). After the silversides, yellowfin goby comprised the highest proportion of dry fish mass (16.9%), followed by longjaw mudsucker (13.6%), staghorn sculpin (7.8%), three-spined stickleback (5.7%), northern anchovy (5.0%), Pacific herring (3.7%), and perches (2.3%). Rainwater killifish and other species of goby each comprised < 2% of dry fish mass. Other species or fish that were unable to be identified comprised 3.0% of dry fish mass.

The most prevalent size class (dry standard length) of fish delivered to tern colonies was 50–75 mm (52.1%), followed by 25–50 mm (28.8%), and then 75–100 mm (16.9%). Fish > 100 mm comprised only 1.7% and fish < 25 mm comprised only 0.5% of all fish recovered at tern colonies. Among species, northern anchovy had the greatest mean (± SD) dry standard length (72 ± 12 mm), followed by staghorn sculpin (71 ± 12 mm), yellowfin goby (70 ± 16 mm), longjaw mudsucker (66 ± 16 mm), silversides (64 ± 13 mm), perches (63 ± 13 mm), Pacific herring (55 ± 10 mm), other gobies (52 ± 22 mm), three-spined stickleback (39 ± 7 mm), and rainwater killifish (31 ± 7 mm; Table 2).

**Table 2:** Sample size, dry standard length (SL; mm), and dry mass (mass; g) of individual fish returned to Forster’s tern (*Sterna forsteri*) colonies in south San Francisco Bay during 2005–2015 by colony and species. The other category includes additional species of fish and invertebrates. Refer to Fig 1 for locations of individual colonies.

					25th	75th			25th	75th
			mean	sd	quantile	quantile	mean	sd	quantile	quantile
Colony	Species	n	SL	SL	SL	SL	mass	mass	mass	mass
A1	Longjaw mudsucker	21	70	17	60	82	1.32	1.01	0.55	1.84
	Northern anchovy	86	73	9	65	78	1.07	0.43	0.78	1.24
	Pacific herring	185	52	9	47	53	0.44	0.38	0.22	0.47
	Perches	11	61	20	43	73	2.3	1.98	0.52	3.57
	Rainwater killifish	23	29	8	22	35	0.18	0.15	0.05	0.27
	Staghorn sculpin	52	74	11	66	81	2.17	1.13	1.38	2.93
	Silversides	818	63	12	55	70	0.85	0.7	0.46	1.05
	Three-spined stickleback	129	42	8	37	47	0.4	0.27	0.23	0.5
	Yellowfin goby	121	70	19	59	83	1.63	1.35	0.68	2.08
	Other gobies	24	72	25	56	96	1.86	1.63	0.54	3.4
	Other	32	73	38	51	70	1.34	1.23	0.61	1.74
A5	Longjaw mudsucker	2	82	0	82	82	0.53	0.42	0.38	0.68
	Northern anchovy	4	72	15	60	83	1	0.59	0.51	1.4
	Pacific herring	1	68	NA	68	68	0.93	NA	0.93	0.93
	Rainwater killifish	2	30	5	28	31	0.18	0.15	0.13	0.24
	Silversides	202	56	9	51	60	0.47	0.28	0.29	0.55
	Three-spined stickleback	63	42	4	40	44	0.37	0.12	0.29	0.43
	Other	1	48	NA	48	48	0.27	NA	0.27	0.27
A7	Longjaw mudsucker	242	64	16	53	76	1.35	1.12	0.6	1.78
	Northern anchovy	27	75	10	69	80	1.47	1.1	0.91	1.61
	Pacific herring	118	57	11	49	63	0.67	0.48	0.35	0.87
	Perches	14	60	8	56	66	1.75	0.74	1.27	2.39
	Rainwater killifish	14	35	5	32	37	0.29	0.12	0.24	0.33
	Staghorn sculpin	65	69	13	60	75	1.74	1.15	0.99	1.99
	Silversides	432	65	12	57	74	0.95	0.7	0.53	1.17
	Three-spined stickleback	454	39	6	34	42	0.32	0.16	0.2	0.41
	Yellowfin goby	210	67	16	55	78	1.39	0.99	0.64	1.84
	Other gobies	50	37	10	33	38	0.21	0.44	0.1	0.18
	Other	102	57	8	55	55	1.1	0.71	0.98	0.98
A8	Longjaw mudsucker	17	77	13	66	86	2.22	1.11	1.53	2.97
	Northern anchovy	8	66	7	61	70	0.9	0.36	0.66	1.15
	Pacific herring	48	57	10	51	62	0.72	0.49	0.41	0.95
	Perches	1	66	NA	66	66	3.21	NA	3.21	3.21
	Rainwater killifish	2	40	11	36	44	0.79	0.31	0.68	0.9
	Staghorn sculpin	10	76	12	70	80	2.22	1.38	1.48	2.46
	Silversides	120	69	17	60	77	1.22	1.03	0.63	1.34
	Three-spined stickleback	19	43	9	38	49	0.41	0.25	0.26	0.53
	Yellowfin goby	28	73	17	61	84	1.63	1.02	0.88	2.23
	Other	15	55	13	52	55	1.3	0.93	0.98	1.41
A12	Longjaw mudsucker	1	80	NA	80	80	2	NA	2	2
	Pacific herring	2	48	7	46	51	0.89	0.15	0.84	0.94
	Staghorn sculpin	2	75	8	72	78	2.82	1.18	2.4	3.23
	Silversides	1	54	NA	54	54	0.86	NA	0.86	0.86
	Three-spined stickleback	1	29	NA	29	29	0.16	NA	0.16	0.16
A16	Longjaw mudsucker	413	67	16	56	77	1.73	1.25	0.81	2.36
	Northern anchovy	63	76	13	70	78	1.32	0.77	0.98	1.25
	Pacific herring	72	54	8	50	56	0.52	0.3	0.33	0.64
	Perches	17	63	9	53	70	2.33	1.23	1.03	3.26
	Rainwater killifish	50	30	6	28	33	0.17	0.12	0.09	0.21
	Staghorn sculpin	72	72	11	66	79	2.11	0.98	1.42	2.63
	Silversides	570	66	10	59	72	0.9	0.48	0.58	1.14
	Three-spined stickleback	876	37	6	32	41	0.23	0.12	0.14	0.29
	Yellowfin goby	280	72	13	65	79	1.74	0.95	1.01	2.2
	Other gobies	73	60	22	39	77	1.37	1.29	0.18	2.17
	Other	26	59	15	48	67	1.23	1.12	0.51	1.74
A2W	Longjaw mudsucker	25	67	14	56	73	1.39	0.96	0.81	1.44
	Northern anchovy	56	69	16	58	80	1.12	0.7	0.64	1.25
	Pacific herring	63	59	12	50	68	0.9	0.55	0.5	1.13
	Perches	9	58	10	50	65	1.89	1.49	0.72	2.18
	Rainwater killifish	10	36	7	29	42	0.24	0.12	0.13	0.35
	Staghorn sculpin	46	69	13	62	77	1.77	0.71	1.28	2.15
	Silversides	751	64	13	56	71	0.91	0.61	0.52	1.14
	Three-spined stickleback	54	46	8	40	51	0.43	0.22	0.26	0.57
	Yellowfin goby	146	70	19	59	80	1.49	0.98	0.76	2.06
	Other gobies	6	39	5	35	43	0.22	0.08	0.19	0.28
	Other	13	65	31	40	78	1.53	1.14	0.62	1.64
AB1	Longjaw mudsucker	28	70	16	61	80	1.97	1.13	1.06	2.72
	Northern anchovy	97	71	12	65	79	1.08	0.63	0.68	1.24
	Pacific herring	73	59	10	51	63	0.75	0.48	0.42	0.85
	Perches	21	59	13	48	67	1.6	1.21	0.67	1.8
	Rainwater killifish	3	44	6	43	48	0.41	0.07	0.37	0.45
	Staghorn sculpin	111	71	9	65	76	1.88	0.7	1.4	2.2
	Silversides	631	66	13	57	73	0.95	0.72	0.49	1.16
	Three-spined stickleback	161	41	5	38	44	0.44	0.26	0.27	0.55
	Yellowfin goby	202	69	15	59	79	1.5	0.98	0.83	1.96
	Other gobies	6	41	9	35	39	0.32	0.29	0.19	0.27
	Other	15	57	27	42	66	0.84	0.69	0.4	1.2
AB2	Longjaw mudsucker	3	80	22	73	93	4.09	2.82	2.53	5.29
	Northern anchovy	13	64	10	56	70	0.81	0.36	0.56	0.99
	Pacific herring	2	54	3	53	55	0.59	0.02	0.58	0.59
	Perches	5	83	4	81	85	3.86	0.49	3.5	4.14
	Staghorn sculpin	15	65	15	57	77	1.96	1.01	1.14	2.77
	Silversides	268	59	11	53	65	0.75	0.44	0.53	0.86
	Three-spined stickleback	9	43	9	38	50	0.37	0.21	0.23	0.4
	Yellowfin goby	19	72	25	50	87	1.71	1.31	0.9	2.27
	Other gobies	1	39	NA	39	39	0.14	NA	0.14	0.14
	Other	4	58	22	40	73	1.83	1.4	1.19	2.18
E2	Longjaw mudsucker	3	81	19	72	91	2.5	1.74	1.52	3.18
	Northern anchovy	5	61	14	61	72	1.03	0.34	0.9	1.3
	Pacific herring	2	62	7	60	65	0.9	0.31	0.79	1
	Perches	1	44	NA	44	44	0.44	NA	0.44	0.44
	Rainwater killifish	27	26	2	25	27	0.05	0.02	0.04	0.06
	Staghorn sculpin	6	71	6	69	74	1.78	0.31	1.54	2.04
	Silversides	109	59	13	51	67	0.66	0.51	0.33	0.77
	Yellowfin goby	4	70	11	66	74	1.16	0.55	1	1.39
E4	Staghorn sculpin	1	73	NA	73	73	2.52	NA	2.52	2.52
	Silversides	4	73	9	66	78	1.52	1	1.08	2.11
E7	Longjaw mudsucker	4	72	17	63	75	2.2	1.58	1.32	2.45
	Northern anchovy	7	76	7	78	79	1.18	0.52	0.81	1.46
	Pacific herring	3	66	0	66	66	1.12	0.93	0.59	1.41
	Perches	2	64	4	62	65	2.16	0.55	1.97	2.36
	Rainwater killifish	2	33	3	32	34	0.22	0.09	0.19	0.25
	Staghorn sculpin	1	59	NA	59	59	1.39	NA	1.39	1.39
	Silversides	54	66	14	57	67	1.1	1.06	0.5	1.44
	Three-spined stickleback	6	41	1	40	42	0.42	0.05	0.38	0.45
	Yellowfin goby	8	55	19	41	65	0.88	0.86	0.28	1.27
	Other	2	44	5	42	46	0.64	0.45	0.48	0.8
E8A	Northern anchovy	1	74	NA	74	74	0.75	NA	0.75	0.75
	Pacific herring	2	76	9	72	79	1.38	0.74	1.12	1.64
	Perches	1	51	NA	51	51	0.89	NA	0.89	0.89
	Silversides	15	74	18	66	87	1.53	0.88	0.89	2.1
	Yellowfin goby	1	53	NA	53	53	0.54	NA	0.54	0.54
E10	Northern anchovy	7	71	8	67	77	1.2	0.43	0.9	1.44
	Perches	19	70	10	64	77	2.69	0.87	1.95	3.24
	Rainwater killifish	3	32	2	31	33	0.24	0.11	0.18	0.29
	Staghorn sculpin	8	58	9	49	65	1.15	0.4	0.92	1.42
	Silversides	28	77	17	67	88	1.67	1.15	0.93	2.26
	Three-spined stickleback	3	42	2	41	43	0.45	0.09	0.4	0.49
	Yellowfin goby	8	80	25	66	94	3.03	1.02	2.59	3.85
NCM	Longjaw mudsucker	2	90	9	86	93	2.78	0.44	2.63	2.94
	Northern anchovy	1	50	NA	50	50	0.3	NA	0.3	0.3
	Pacific herring	1	43	NA	43	43	0.31	NA	0.31	0.31
	Perches	1	71	NA	71	71	3.1	NA	3.1	3.1
	Staghorn sculpin	1	29	NA	29	29	1.48	NA	1.48	1.48
	Silversides	45	66	14	56	74	1.22	0.83	0.54	1.85
	Three-spined stickleback	4	29	6	26	30	0.1	0.08	0.05	0.12
	Yellowfin goby	7	68	12	61	76	1.37	0.61	1.01	1.73
	Other	3	52	18	42	59	1.41	0.4	1.3	1.64
N7	Longjaw mudsucker	61	59	13	48	69	1.39	1.01	0.6	2.01
	Northern anchovy	4	93	16	86	95	2.31	1.12	1.8	2.43
	Pacific herring	4	53	4	50	54	0.41	0.08	0.35	0.47
	Perches	1	63	NA	63	63	2.37	NA	2.37	2.37
	Rainwater killifish	4	29	4	27	31	0.18	0.09	0.13	0.22
	Staghorn sculpin	3	65	19	59	76	1.57	1.13	1.15	2.21
	Silversides	67	74	23	53	90	1.74	1.38	0.46	2.27
	Three-spined stickleback	76	40	5	38	43	0.29	0.11	0.2	0.36
	Yellowfin goby	2	64	5	62	65	1.05	0.49	0.88	1.23
	Other gobies	9	40	6	36	44	0.36	0.2	0.23	0.5
	Other	39	55	9	53	55	1.03	0.68	0.85	0.98
R1	Longjaw mudsucker	8	57	10	53	56	1.08	0.76	0.68	1.18
	Northern anchovy	40	73	11	65	80	1.11	0.45	0.9	1.22
	Rainwater killifish	5	39	4	38	40	0.44	0.07	0.39	0.46
	Staghorn sculpin	2	85	36	72	97	2.09	0.29	1.98	2.19
	Silversides	73	82	25	66	99	2.15	1.68	0.94	3.21
	Yellowfin goby	7	80	11	78	88	2.67	1.19	2.31	3.15
	Other gobies	2	32	10	29	36	0.16	0.05	0.14	0.17
	Other	1	69	NA	69	69	2.62	NA	2.62	2.62
All	Longjaw mudsucker	830	66	16	55	77	1.60	1.21	0.74	2.14
	Northern anchovy	419	72	12	65	79	1.15	0.66	0.77	1.30
	Pacific herring	576	55	10	49	61	0.62	0.47	0.31	0.79
	Perches	103	63	13	55	71	2.18	1.29	1.14	3.12
	Rainwater killifish	145	31	7	26	35	0.19	0.16	0.06	0.28
	Staghorn sculpin	395	71	12	64	77	1.92	0.94	1.29	2.33
	Silversides	4188	64	13	56	72	0.93	0.74	0.48	1.14
	Three-spined stickleback	1855	39	7	35	42	0.30	0.18	0.18	0.37
	Yellowfin goby	1043	70	16	60	80	1.57	1.05	0.82	2.11
	Other gobies	171	52	22	36	70	0.95	1.24	0.14	1.52
	Other	253	59	19	55	61	1.16	0.89	0.78	1.09

### Relative prey species abundance and dry mass composition among colonies

Overall, the relative abundances of prey fish species among seven repeatedly-sampled tern colonies (A1, A7, A8, A16, A2W, AB1, and AB2 colonies; Fig 1) were more different than would be expected by chance (*A* = 0.12, *p* = 0.001). Among colonies, the relative fish species abundance was the most consistent among years at A2W (δ = 0.32) and A1 (δ = 0.32), followed by AB2 (δ = 0.34). Notably, each of these three tern colonies are located adjacent to each other in the Moffett pond complex. The relative prey fish species abundance at A8, a colony located in Alviso, was the least consistent among years (δ = 0.45; Table 3). Comparing individual colonies, five of the colonies (A1, A2W, AB1, AB2, and A8) were as similar to each other as would be expected by chance, based on the variability observed within each colony (Table 3). In contrast, the two remaining colonies from the Alviso pond complex, A7 and A16, were both less similar to the A1, A2W, AB1, and AB2 colonies than would be expected by chance (Table 3). The A8 colony was not distinguishable from the A7 or A16 colonies (Table 3), all of which were located in the Alviso pond complex.

**Table 3:** Results from multi-response permutation procedure (mrpp) analysis comparing species relative abundance (top) and the species composition of dry mass (bottom) for fish dropped at Forster’s tern (*Sterna forsteri*) colonies in south San Francisco Bay during 2005–2015.

Colony	A1	A16	A2W	A7	A8	AB1	AB2
Relative species abundance							
A1	**δ = 0.32**						
A16	*0.*48*, *0*.*16*, *0*.*007*	**δ = 0.39**					
A2W	0.30, -0.03, 0.89	*0.*51*, *0*.*19*, *0*.*003*	**δ = 0.32**				
A7	*0.*47*, *0*.*14*, *0*.*003*	0.36, -0.04, 0.96	*0.*48*, *0*.*16*, *0*.*002*	**δ = 0.39**			
A8	0.40, 0.02, 0.28	0.47, 0.06, 0.13	0.41, 0.03, 0.16	0.43, 0.01, 0.33	**δ = 0.45**		
AB1	0.35, -0.03, 0.89	*0.*53*, *0*.*14*, *0*.*014*	0.34, -0.04, 0.86	*0.*49*, *0*.*10*, *0*.*017*	0.43, -0.01, 0.56	**δ = 0.42**	
AB2	0.33, < 0.00, 0.44	*0.*57*, *0*.*24*, *0*.*021*	0.32, -0.01, 0.50	*0.*55*, *0*.*21*, *0*.*006*	0.47, 0.09, 0.08	0.36, -0.02, 0.64	**δ = 0.34**
Dry mass composition							
A1	**δ = 0.33**						
A16	*0.*50*, *0*.*20*, *0*.*003*	**δ = 0.35**					
A2W	0.30, -0.02, 0.77	*0.*51*, *0*.*23*, *0*.*003*	**δ = 0.30**				
A7	*0.*43*, *0*.*09*, *0*.*006*	0.38, 0.01, 0.31	*0.*43*, *0*.*11*, *0*.*006*	**δ = 0.40**			
A8	0.42, 0.03, 0.20	0.49, 0.10, 0.08	0.42, 0.05, 0.12	0.42, < -0.01, 0.55	**δ = 0.46**		
AB1	0.38, -0.03, 0.77	*0.*55*, *0*.*15*, *0*.*009*	0.37, -0.02, 0.69	0.47, 0.05, 0.082	0.48, 0.01, 0.33	**δ = 0.47**	
AB2	0.39, < -0.01, 0.44	*0.*58*, *0*.*20*, *0*.*021*	0.37, -0.02, 0.61	*0.*50*, *0*.*10*, *0*.*021*	0.50, 0.05, 0.12	0.44, -0.02, 0.66	**δ = 0.45**

Intra-colony comparisons are in bold on the diagonal with the weighted within-group distance (δ value).

Inter-colony comparisons are shown in the other cells (δ value, *A* statistic, and *p* value), with significant values shown in italics with an *.

Similar to relative fish abundance, the dry mass composition at repeatedly-sampled Forster’s tern colonies was also more different than would be expected by chance (*A* = 0.10, *p* = 0.002). The dry mass composition was most consistent among years at the A2W (δ = 0.30), A1 (δ = 0.33), and A16 colonies (δ = 0.35; Table 3). Similar to the relative species abundances, five of the colonies (A1, A2W, AB1, AB2, and A8) had dry mass compositions as similar to each other as would be expected by chance (Table 3). The dry mass compositions at the A16 and A7 colonies were both less similar to the A1, A2W, and AB2 colonies than would be expected by chance (Table 3). A16 was also less similar to AB1 than would be expected by chance (*A* = 0.14, *p* = 0.014). However, unlike for relative species abundance, the dry mass composition at A7 was marginally indistinguishable from AB1 (*A* = 0.05, *p* = 0.08). In addition, the A8 colony was not different from the A7 or A16 colonies (Table 3).

### Relative prey species abundance, relative mass, and size trends over time

**Fig 4:**
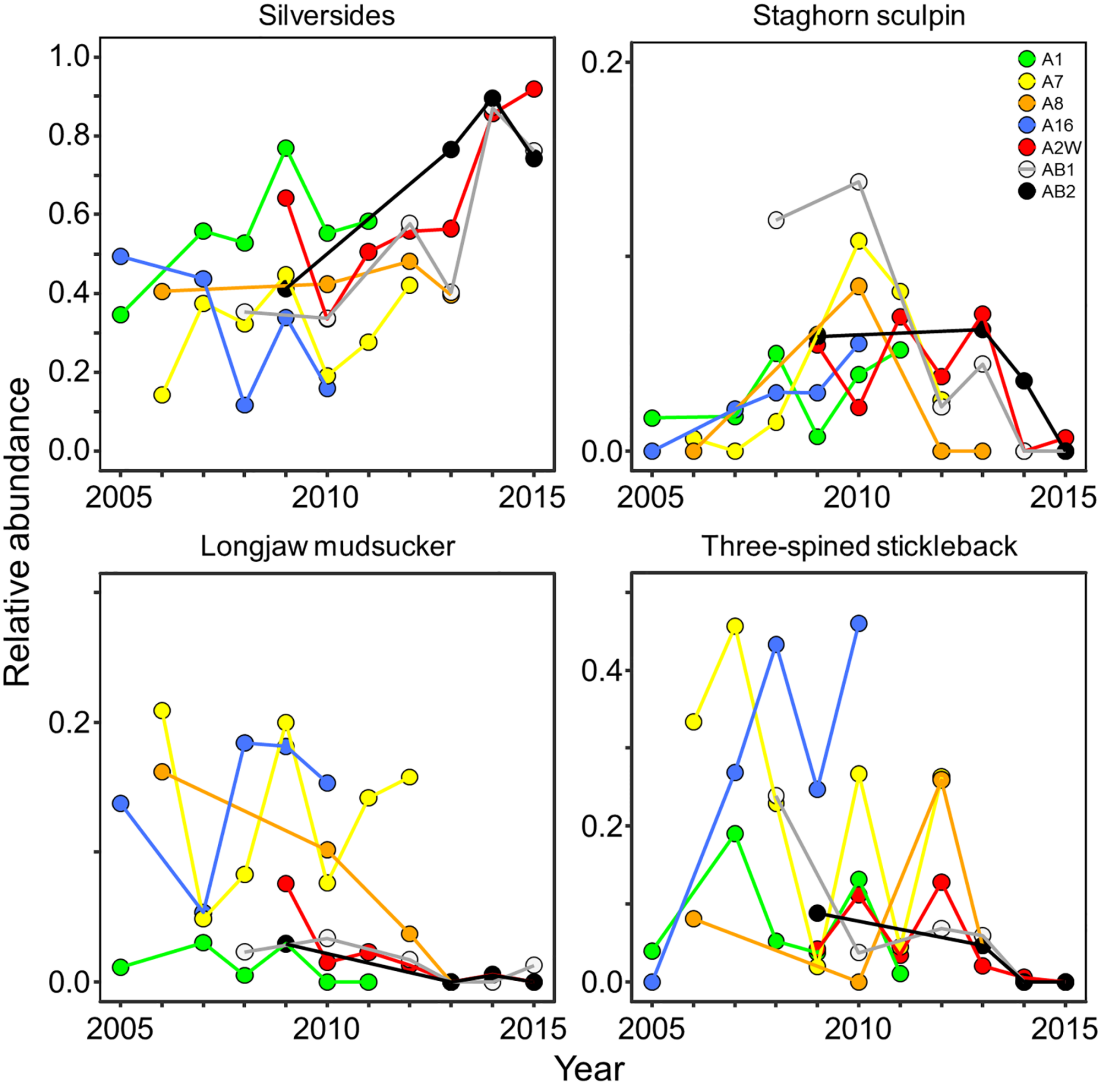
Relative abundance of four species of fish returned to Forster’s tern (*Sterna forsteri*) colonies. Forster’s tern breeding colonies were sampled in south San Francisco Bay, California during 2005–2015. Temporal trends for four main prey species of Forster’s terns are color-coded by colony. Silversides (*Menidia audens* and *Atherinops affis*) increased in relative abundance over time, whereas longjaw mudsucker (*Gillichthys mirabilis*) and three-spined stickleback (*Gasterosteus aculeatus*) decreased over time. Trends of staghorn sculpin (*Leptocottus armatus*) relative abundance varied among colonies. Not every location had a breeding colony of Forster’s terns each year.

The relative abundance of certain prey species changed from 2005 to 2015 at some Forster’s tern colonies in San Francisco Bay (MANOVA: *F*_42,144_ = 1.50, *p* = 0.04, Pillai’s trace = 1.82; Fig 4; see Table 4 for statistical output from subsequent ANOVAs). For silversides, the effect of year differed among colonies (*F*_6,25_ = 2.56, *p* = 0.05). The A16 colony was the only colony that had a marginally negative slope coefficient (-0.31 ± 0.17 standard error; *t* = -1.81, df = 25, *p* = 0.08). Given the slope coefficient and the substantial restoration actions that occurred at this colony at the start of our study that increased tidal exchange and decreased salinity [24], we removed A16 and reran the model. When we reran the model without A16, the year × colony interaction was no longer significant, and we observed an overall increase in the relative abundance of silversides returned to Forster’s tern colonies from 2005 to 2015. The relative abundances of silversides were similar among colonies, although one Moffett colony (A1) had ≥ 1.5 times the relative abundance of silversides than at the A7 and A8 Alviso colonies (*t* ≥ 2.10, df = 27, *p* ≤ 0.05). Run separately, there was not enough evidence to show a change in the relative abundance of silversides at A16 over time (*F*_1,3_ = 2.58, *p* = 0.21). For stickleback, the significant interaction (*F*_6,25_ = 3.04, *p* = 0.02) was also being driven by A16, as it had a positive slope coefficient that was significantly different than all of the other colonies (*t* ≥ 2.18, df = 25, *p* ≤ 0.04). Overall, the relative abundance of stickleback decreased when we excluded A16. In contrast, stickleback showed a marginally non-significant increase at A16 (*F*_1,3_ = 7.72, *p* = 0.07). The relative abundance of longjaw mudsucker decreased at all colonies from 2005 to 2015 and varied among colonies. With the exception of A8, the mean relative abundance of longjaw mudsucker at the Alviso colonies was ≥ 4.3 times greater than at the Moffett colonies (*t* ≥ 2.91, df = 31, *p* ≤ 0.007). Additionally, the mean relative abundance of mudsucker at A8 was ≥ 3.1 times greater than at two of the Moffett colonies, AB2 and A1 (*t* ≥ 2.14, df = 31, *p* ≤ 0.04). We did not observe any overall increase or decrease in the relative abundance of Pacific herring or northern anchovy over time, although the relative abundance of northern anchovy was ≥ 3.6 times greater at two of the Moffett colonies (A1 and AB1) than the Alviso colonies (*t* ≥ 2.18, df = 31, p ≤ 0.04). The relative abundance of staghorn sculpin had less clear overall temporal trends, with an increase at one Alviso colony (A7; *t* = 2.38, df = 25, *p* = 0.03) and a decrease at one Moffett colony (AB1; *t* = -3.21, df = 25, *p* = 0.004). There were no clear temporal trends for staghorn sculpin at the other colonies.

**Table 4:** Statistical results from univariate ANOVAs, following a MANOVA, to examine the relative abundance and relative biomass of seven species groups in the diet of Forster’s tern (*Sterna forsteri*) at repeatedly sampled colonies in south San Francisco Bay during 2005–2015. The model for each species was first run with a year × colony interaction. We removed any non-significant interactions and reran the model to test the effect of year and colony. Significant effects are bold and italicized.

	Interaction		Interaction removed			
Species	F_6,25_	*p*	Year *F*_1,31_	Year *p*	Colony *F*_6,31_	Colony *p*
Relative abundance						
Silversides^a^	1.38	0.27	***17*.*13***	***< 0*.*001***	***2*.*98***	***0*.*03***
Longjaw mudsucker	2.17	0.08	***14*.*87***	***< 0*.*001***	***9*.*22***	***< 0*.*001***
Three-spined stickleback^a^	0.98	0.45	***10*.*07***	***0*.*004***	1.05	0.41
Pacific herring	1.27	0.31	0.37	0.55	1.25	0.31
Northern anchovy	1.78	0.14	< 0.01	0.97	***2*.*50***	***0*.*043***
Staghorn sculpin	***4*.*11***	***0*.*005***	---	---	---	---
Yellowfin goby	1.85	0.13	0.02	0.88	0.56	0.76
Relative biomass						
Silversides	2.09	0.09	***8*.*87***	***0*.*006***	1.56	0.19
Longjaw mudsucker	1.18	0.35	***12*.*98***	***0*.*001***	***3*.*87***	***0*.*005***
Three-spined stickleback^a^	1.06	0.41	***8*.*75***	***0*.*006***	0.54	0.74
Pacific herring	1.71	0.16	0.21	0.65	1.61	0.18
Northern anchovy	1.73	0.15	0.04	0.84	2.14	0.08
Staghorn sculpin	***3*.*24***	***0*.*02***	---	---	---	---
Yellowfin goby	***2*.*59***	***0*.*04***	---	---	---	---

^a^ The models for silversides and three-spined stickleback were rerun without the A16 colony, after it was determined that A16 was causing the significant year × colony interaction. The degrees of freedom for the *F*-statistics with and without the interaction, after removing A16, were 5,22 and 5,27, respectively.

Similar to relative abundance, the relative dry mass of some prey species changed from 2005 to 2015 at Forster’s tern colonies in San Francisco Bay (*F*_42,144_ = 1.47, *p* = 0.05, Pillai’s trace = 1.820; Table 4). Overall, the relative mass of silversides increased from 2005 to 2015, while the relative mass of longjaw mudsucker decreased. For stickleback, a significant year × colony interaction (*F*_6,25_ = 2.50, *p* = 0.05) was being driven by A16, as it had a positive slope coefficient (*t* = 2.31, df = 25, *p* = 0.03). When A16 was removed, the relative mass of stickleback decreased from 2005 to 2015. Run separately, stickleback showed a marginally non-significant increase at A16 (*F*_1,3_ = 7.45, *p* = 0.07). Whereas there were no overall differences in the relative mass of silversides and stickleback among colonies, the three Alviso colonies had ≥ 15 times the relative mass of longjaw mudsucker than at the A1 colony in Moffett (*t* ≥ 2.62, df = 31, *p* ≤ 0.01). For both yellowfin goby and staghorn sculpin, we observed an increase in the relative mass at A16 (*t* ≥ 2.13, df = 25, *p* ≤ 0.04). Additionally, yellowfin goby increased at AB2 (*t* = 2.25, df = 25, *p* = 0.02) and staghorn sculpin declined at AB1 (*t* = -2.99, df = 25, *p* = 0.006). We did not observe any overall temporal trends in relative dry mass for northern anchovy or Pacific herring or differences among colonies.

Several fish species returned to Forster’s tern colonies changed in size from 2005 to 2015, with most changes suggestive of an overall decrease in fish body size. The average staghorn sculpin decreased annually by 1.8 mm in standard length (*F*_1,358_ = 30.49, *p* < 0.001) and 6.6% in dry mass (*F*_1,364_ = 20.75, *p* < 0.001). Similarly, northern anchovy decreased annually by 1.0 mm in standard length (*F*_1,45.9_ = 8.65, *p* = 0.005) and 3.2% in dry mass (*F*_1,72.2_ = 4.70, *p* = 0.03). Silversides, the most commonly observed species group returned to Forster’s tern colonies, decreased in length annually by 0.2 mm (*F*_1,1774.2_ = 4.51, *p* = 0.03) but did not change in dry mass (silversides: *F*_1,653.7_ = 2.64, *p* = 0.10). In contrast, three-spined stickleback did not change in standard length (*F*_1,1463.5_ = 0.45, *p* = 0.50) but decreased annually by 5.3% in dry mass (*F*_1,1485.2_ = 20.02, *p* < 0.001). Yellowfin goby was the only species that demonstrated an increase in size, by 1.1 mm/yr in standard length (*F*_1,265.8_ = 15.47, *p* < 0.001) and 4.8% annually in dry mass (*F*_1,342.9_ = 11.62, *p* < 0.001). Longjaw mudsucker and Pacific herring did not change in standard length (mudsucker: *F*_1,608.0_ = 3.47, *p* = 0.06; herring: *F*_1,122.3_ = 0.32, *p* = 0.58) or dry mass (mudsucker: *F*_1,681.5_ = 3.00, *p* = 0.08; herring: *F*_1,333.4_ < 0.01, *p* = 0.98).

## Discussion

Silversides (Mississippi silverside and topsmelt silverside; family Atherinopsidae) were the predominant prey species returned to Forster’s tern colonies in San Francisco Bay over an 11-year period. Silversides comprised more than twice that of the next most common species, both by relative abundance of individuals (42%) and relative dry mass (40%). Three-spined stickleback were the second most common species returned to Forster’s tern colonies (19% relative abundance) over the entire study. Previous fish sampling studies revealed that topsmelt silverside were present in all sampled ponds and sloughs in south San Francisco Bay, and comprised the majority of fish collected in gillnets from 2004–2006, while three-spined stickleback were the most abundant fish species sampled using minnow traps in ponds sampled in 2006 [20,21]. These observations suggest that silversides and three-spined stickleback were two of the most abundant Forster’s tern prey items at the start of our study, and they were the two most abundant species groups returned to Forster’s tern colonies. However, the relative contribution of three-spined stickleback was markedly lower when examined using the relative dry mass by species (6%) because of their small size. Instead, yellowfin goby (17% by mass and 10% by abundance) and longjaw mudsucker (14% by mass and 8% by abundance) contributed more by dry mass.

The prey species returned to Forster’s tern colonies had similarities with coastally-foraging California least terns (*Sterna antillarum browni*), where 70% of the diet was comprised of silversides and northern anchovy [12]. No other comparable studies exist for Forster’s terns, as previous foraging-related research on Forster’s terns was either conducted inland [23], on the Atlantic coast [25], or was primarily focused on the hunting behavior of Forster’s terns and not on estimates of diet or prey selection [34]. However, Atlantic silverside (*Menidia menidia*) were suggested to be an important prey item of Forster’s terns on the Atlantic coast, as they comprised 99.6% of samples collected in a seine adjacent to observations of foraging terns [25]. Inland, yellow perch (*Perca flacescens*) and shiner (*Notropis* spp.) were the most important species in courtship and chick feedings by Forster’s terns, and these two species groups comprised 97% of species collected in seines; Atherinopsidae was not represented by any species in this study [23]. The species composition of observed chick feeding events and the prey species returned to and dropped on breeding colonies were relatively similar for several studies on other tern species, although the proportions of larger bodied prey species were slightly elevated in the sample of prey species dropped on the colony [11–13]. It is unknown how the fish species returned to Forster’s tern colonies in the present study relates to the fish species consumed by Forster’s tern adults or their chicks. However, based on previous studies, the major prey species determined using fish returned to and dropped on the colony were the same as those ingested by chicks or fed to mates [11–13]. All methods used to estimate avian diet have significant challenges and limitations, including the cost, feasibility of direct observation, and level of invasiveness. In spite of the limitations of our sampling method, collecting fish returned to and dropped on the colony provided an extensive amount of inexpensive and consistently collected data over an 11-year period.

Tern foraging behavior typically consists of plunge-diving from a stationary hovering position [34], allowing them to capture fish present either in shallow water or in the upper portion of the water column in deeper water. Consequently, silversides are vulnerable to predation by Forster’s terns because of their surface-dwelling and schooling behaviors [35]. In contrast, yellowfin goby and longjaw mudsucker, which both comprised a lower proportion of fish returned to Forster’s tern colonies, are more demersal species [35] and likely are only accessible to Forster’s terns when they are in shallow water.

We observed temporal differences in the fish returned to Forster’s tern colonies in San Francisco Bay over the 11-year sampling period, suggesting relatively localized changes in relative prey availability and the size of some prey species. Generally, the relative abundance and dry mass of silversides increased over time. Additionally, the average silverside decreased in size by 1.2 mm over our study but did not change in mass. Concurrently, we observed a decrease in the overall relative abundance and dry mass of three-spined stickleback and longjaw mudsucker returned to tern colonies. In contrast to silversides, the average three-spined stickleback decreased in dry mass by 5.3% annually (14 mg dry mass or 46 mg wet weight, based on a moisture content of 69.5% [7]) but did not decrease in length, suggesting that stickleback may have decreased in condition over time. Salt ponds and sloughs in south San Francisco Bay contain the species we collected at Forster’s tern colonies [20,21]. However, salt ponds typically contained a smaller subset of the fish species observed in adjacent sloughs [21], and salinity was the most important environmental variable related to the spatial distribution of species [20]. Restoration of tidal exchange to three previously isolated salt ponds decreased pond salinity from levels intolerant to fish and allowed more salt-tolerant fish species (*e*.*g*., topsmelt silverside, northern anchovy, and longjaw mudsucker) to colonize, which resulted in a salt-related gradient of species observed from the upstream reaches of the sloughs down to the saltier ponds [20]. Furthermore, an unplanned breach of levees in North San Francisco Bay caused a marked decrease in longjaw mudsucker as the community composition in a formerly hypersaline pond shifted from mostly salt-tolerant species to a species assemblage that included some freshwater fish [20]. Consequently, the changes we observed in relative fish abundance returned to Forster’s tern colonies over the course of our study could be a result of changes in prey selection or may be the result of changes in fish availability because of altered habitat from management associated with the South Bay Salt Pond Restoration Project (www.southbayrestoration.org).

Breeding Forster’s terns are central place foragers that tend to feed within 6.2 km of their breeding colony [19]; therefore, the inter-colony differences we observed in relative species abundance and dry mass composition suggested heterogeneity in the available prey base among colonies. Geographically, the colonies with the most consistent relative species abundance over time were located adjacent to each other in the Moffett pond complex (A1, A2W, AB1, and AB2) and were separated from the three additional repeatedly-sampled colonies in the Alviso pond complex (A7, A8, and A16; Fig 1). The A7 and A16 colonies were distinguishable from the other colonies, both in relative species abundance as well as dry mass composition, which appeared to be generally driven by a greater proportion of longjaw mudsucker returned to the Alviso colonies and a greater proportion of northern anchovy returned to the Moffett colonies. The high variability of relative species abundances among years at the A8 colony made it indistinguishable overall from any other colony.

Two of the repeatedly sampled colonies stood apart from the others in terms of the intra-colony species heterogeneity (A8) and temporal trends (A16), which may have been a direct result of altered management practices at those sites or differences in available foraging habitat. Previously, breaching of levees in the south and north regions of San Francisco Bay caused changes in species assemblages as connectivity between habitats was increased and salinity levels changed [20,21]. In our study, the A8 colony, sampled in 2006, 2010, 2012, and 2013, had the highest intra-colony variability in relative species abundance. Furthermore, A8 had relatively low sample sizes of fish returned to the colony, which may have contributed to the observed variability. However, as part of the South Bay Salt Pond Restoration Project, the habitat in A8 changed markedly after the 2010 Forster’s tern breeding season, at which point in time managers physically interconnected the pond to two other managed ponds, and the water depth of the entire complex was increased. Furthermore, the levees for an adjacent and previously dry pond bed (A6) were breached in the fall of 2010 to allow for the development of tidal marsh habitat, providing new habitat for fish within the foraging range of the A8 colony. Thus, two of the sampling years for the A8 colony occurred before these management actions and two sampling years occurred after, which may have contributed to the substantial heterogeneity observed in the relative species abundance over time. Management actions for A16 as part of the South Bay Salt Pond Restoration Project increased tidal exchange and decreased salinity [24], which may have contributed to the temporal trends that we observed in the fish returned to the A16 colony. Specifically, all silversides collected from A16 in 2005 were identified as topsmelt silverside (49% of fish, *n* = 69), suggesting that Mississippi silverside had not yet colonized this pond. As the salinity decreased in pond A16, this may have allowed other species, such as three-spined stickleback, to colonize and increase in relative abundance over time.

Changes in prey availability and diet could have important implications for Forster’s terns in the San Francisco Bay Estuary, such as reproductive success and contaminant exposure. In particular, mercury contamination is known to be an important issue for waterbirds breeding within the estuary [36]. The highest whole-body mercury concentrations in fish collected across 27 managed wetlands, 3 tidal marshes/sloughs, and 2 open water sites in the San Francisco Estuary were in Mississippi silverside (0.83 ± 0.02 μg/g dw), followed by topsmelt silverside (0.55 ± 0.02 μg/g dw; [7]). Whereas we were unable to separate these two species in our analysis, their combined relative abundance and relative dry mass increased in the diet of Forster’s terns over the past 11 years. Consequently, a temporal shift in diet could cause an increase in mercury exposure and toxicological risk for Forster’s terns. In contrast, species that had significantly lower mercury concentrations than silversides, such as three-spined stickleback (0.45 ± 0.01 μg/g dw) and longjaw mudsucker (0.36 ± 0.01 μg/g dw), were the prey species that declined in relative abundance over time [7].

Forster’s terns breeding in the San Francisco Bay area relied heavily on surface-dwelling silversides and our results showed that their dependence on these fishes has increased. As central place foragers, the fish returned to Forster’s tern colonies suggest that there were differences in relative prey availability among colonies and over time. The abundance of three-spined stickleback and longjaw mudsucker returned to Forster’s tern colonies decreased at multiple colonies, suggesting that their relative availability in the environment also has declined, possibly in response to habitat alteration. Future studies could evaluate if changes in diet among colonies and over time has resulted in differential reproductive success and contaminant exposure.
